# Imipramine-induced immunomodulation and intracellular growth inhibition during *Brucella abortus* 544 infection in RAW 264.7 cells and BALB/c mice

**DOI:** 10.3389/fvets.2025.1598106

**Published:** 2025-06-16

**Authors:** Ched Nicole Turbela Aguilar, Tran Xuan Ngoc Huy, Trang Thi Nguyen, Said Abdi Salad, Seong Eun Cho, Il-Hwa Hong, Wongi Min, Hu Jang Lee, Suk Kim

**Affiliations:** ^1^Institute of Animal Medicine, College of Veterinary Medicine, Gyeongsang National University, Jinju, Republic of Korea; ^2^Institute of Applied Sciences, HUTECH University, Ho Chi Minh City, Vietnam

**Keywords:** *Brucella abortus*, imipramine hydrochloride, BALB/c mouse, RAW 264.7 cells, immunomodulation

## Abstract

Brucellosis is a significant zoonotic infection with increasing global prevalence. Traditional treatments rely on antibiotic combinations, but challenges such as drug resistance and relapse necessitate the exploration of alternative therapeutic options. Imipramine hydrochloride (ImiP) has shown potential as an adjunctive treatment for infectious diseases. This study investigates the immunomodulatory effects of ImiP in *B. abortus* 544 infections in murine macrophages and BALB/c mice. *In vitro*, RAW 264.7 cells exposed to ImiP exhibited reduced *B. abortus* replication, decreased nitrite levels, and enhanced bactericidal effects. *In vivo*, ImiP treatment significantly decreased bacterial loads in the spleen (10 mg/kg, **p < 0.01; 20 mg/kg, *p < 0.05) and liver (10 mg/kg, **p < 0.01; 20 mg/kg, ***p < 0.001), compared to untreated controls. Histopathological analysis revealed minimal liver microgranuloma formation and periportal inflammation in ImiP-treated mice. Moreover, flow cytometry showed decreased CD4^+^ and CD8^+^ T cell expression, while serum cytokine profiling indicated a Th1-driven immune response, characterized by elevated levels of IL-12 and decreased IL-10. These findings suggest that ImiP possesses both immunomodulatory and antibacterial effects, highlighting its potential as an adjunctive therapy for brucellosis.

## 1 Introduction

Brucellosis is a zoonotic disease that has been present for many years and is caused by Gram-negative, facultative intracellular coccobacilli of the genus *Brucella*. Currently, there are 12 *Brucella* species known to infect a wide range of animals. However, the primary species that cause disease in humans are *B. abortus* (cattle), *B. suis* (pigs), and *B. melitensis* (sheep and goats). Brucellosis is considered an emerging and re-emerging disease, with over 500,000 human cases reported annually worldwide (1, 2). The high incidence is attributed to factors such as increasing travel, trade, and migration (3).

Humans acquire the infection through ingestion of unpasteurized milk products and direct contact with infected animals. Symptoms include fever, fatigue, arthritis, and hepatitis (4). Disease transmission in animals occurs through direct contact during mating or exposure to infected blood, placenta and milk. Affected animals may present with abortion, mastitis, stillbirth, and sterility (5). Although brucellosis has a low mortality rate, infection can cause severe complications, leading to significant economic losses (6). Generally, brucellosis is treated with long-term antibiotic therapy. However, a major challenge associated with prolonged antibiotic use is the development of resistance and reduced sensitivity to other antibiotics (7, 8). While live-attenuated vaccines are available for animals, an effective vaccine for humans has yet to be developed (9).

In the search for new treatment options for infectious diseases such as brucellosis, recent studies have focused on the potential of non-antibiotic drugs as adjunctive therapy (10, 11). Imipramine hydrochloride (ImiP) is a tricyclic antidepressant (TCA) used to treat chronic depression. It inhibits serotonin and norepinephrine reuptake by blocking their respective neurotransmitter transporters (12). ImiP has demonstrated diverse biological properties, including anti-cancer, anti-parasitic, anti-inflammatory, and antiviral effects (13–15). It has also been shown to alleviate brucellosis symptoms by improving serotonin levels, muscle strength, and reducing bacterial load in infected mice (16). In this study, we investigated the potential of ImiP as a treatment for *B. abortus* 544 infections in murine macrophages and BALB/c mice, focusing on its immunomodulatory effects, bacterial clearance, and impact on the host immune response.

## 2 Materials and methods

### 2.1 Imipramine preparation

Imipramine hydrochloride (ImiP) (Sigma, I7379) was dissolved in deionized water to prepare a 0.1 M stock solution. The solution was sterilized by filtration through a 0.22 μm pore-size membrane to eliminate impurities and subsequently stored at −20°C. Working solutions were prepared by diluting the stock with sterilized phosphate-buffered saline (PBS).

### 2.2 Cell culture and bacterial growth conditions

RAW 264.7 cells (ATCC, TIB-71) were cultured in RPMI 1640 medium (Gibco, CA, USA, 11875119) supplemented with 10% heat-inactivated fetal bovine serum (FBS) (Gibco, 1600–044), with or without 1% of 100 × penicillin/streptomycin (Gibco, Invitrogen, NY, USA) at 37°C in a 5% CO₂ incubator. For specific assays, cells were seeded in 6-well and 96-well plates at densities of 1 × 10^6^ and 5 × 10^4^ cells per well, respectively, in RPMI medium with 10% FBS. The plates were incubated overnight before use.

The smooth, virulent, wild-type *B. abortus* 544 biovar 1 (ATCC 23448) was cultured on *Brucella* agar at 37°C in a 5% CO₂ atmosphere for 72 h. For cell infection and *in vivo* experiments, one colony was inoculated into *Brucella* broth (BBL BD, San Jose, CA, USA) and incubated at 37°C with continuous shaking for 48 h in a biosafety level 3 (BSL-3) containment facility.

### 2.3 Cell viability assessment assay

RAW 264.7 cells were seeded in 96-well tissue culture plate were treated with different concentrations of ImiP (1,000; 500; 250; 125; 62.5; 31.25; 15.63; 7.81; and 3.91 μM) for 72 h. The cells were washed with PBS then fresh RPMI medium containing 10% FBS and 5 mg/mL MTT (3-(4,5-dimethylthiazol-2-yl)-2,5-diphenyltetrazolium bromide) was added to each well. The plate was wrapped in foil and incubated for 4 h under the same conditions. Thereafter, the medium was removed, 150 μL of dimethyl sulfoxide (DMSO) was added to each well, and the plate was incubated for 15 min. Cell viability was measured at 540 nm using a spectrophotometer (Thermo Labsystems Multiskan, Chantilly, VA, USA).

### 2.4 Bactericidal assay

*B. abortus* 544 at a concentration of approximately 10^4^ colony-forming units (CFUs)/well was incubated with different concentrations of ImiP (37.5, 7.5, and 0.25 mM), with PBS as the control. The plate was incubated for 0, 4, 8, 24, and 48 h at 37°C in a 5% CO₂ atmosphere. At each time point, the mixture was serially diluted in RPMI, plated onto *Brucella* agar, and incubated for 72 h. CFUs were counted to evaluate bacterial survival.

### 2.5 Bacterial internalization and intracellular replication assay

For the bacterial internalization assay, 96-well tissue culture plate seeded with RAW 264.7 cells were pre-treated with ImiP at a concentration of 125 and 250 μM for 6 h before infection with *B. abortus* 544 at a multiplicity of infection (MOI) of 1,000. The plate was centrifuged at 200 × *g* for 5 min and then incubated at 37°C in a 5% CO_2_ atmosphere. At 5, 20, and 60 min post-infection (pi), the cells were treated with gentamicin (50 μg/mL) per well for 30 min. The cells were washed with PBS, lysed in 100 μL distilled water, serially diluted and plated onto *Brucella* agar plates.

For the intracellular replication assay, cells were infected with *B. abortus* for 1 h and treated with ImiP (125 and 250 μM) and gentamicin (50 μg/mL) at 4, 24, and 48 h post-infection (pi). At each time point, the cells were washed, lysed and plated same as described in the internalization assay. CFUs were counted after 3 days to evaluate internalization and intracellular replication efficiency.

### 2.6 Nitrite oxide (NO) measurement by Griess assay

Following the protocol for intracellular growth assay, RAW 264.7 cells were infected with *B. abortus* and treated with ImiP. Cell supernatants were collected at 24 and 48 h pi and nitrite levels were measured using the Griess reagent kit (Promega, Cat# G2930) according to the manufacturer’s instructions.

### 2.7 Mice infection and treatment

Eight-week-old, specific-pathogen-free female BALB/c mice (Samtako Bio Co., Ltd., Korea) were randomly grouped into six groups consisting of six mice each. The mice were acclimatized for a week in a microisolator cage at 23 ± 1°C with a 12 h light/dark cycle, with ad libitum water and feed. Prior to infection, the mice were orally treated with ImiP (10 mg/kg/day or 20 mg/kg/day) or PBS (control) via gavage needle for 7 days. The selected ImiP doses were based on previous studies in mouse models examining its effects on neurological behavior, antidepressant activity, and antibacterial properties (17–20). Three groups of BALB/c mice were intraperitoneally infected with *B. abortus* 544 (5 × 10^6^ CFU in 100 μL PBS), while the remaining three groups received treatment without infection. The treatment continued for 14 days pi, during which all animal groups were monitored for clinical symptoms. On day 14 pi, peripheral blood samples were collected from the tail vein and the mice were sacrificed via cervical dislocation to collect the liver and spleen. The organs were individually weighed, a 1 cm sample was collected and fixed in 10% neutral-buffered formalin for histopathological examination, and a 0.05 g portion of each organ was collected for bacterial proliferation. The organs were homogenized in PBS then the homogenates were serially diluted and plated onto agar incubated at 37°C in a 5% CO_2_ for 3 days. Bacterial CFUs were log₁₀-transformed to calculate protection units. All animal handling and sacrifice procedures were reviewed and approved by the Animal Ethical Committee of Chonbuk National University (Authorization Number CBNU-2021-037).

### 2.8 Measurement of CD4^+^ and CD8^+^ T-cell population

CD4^+^ and CD8^+^ T-cell populations were determined using two-color flow cytometry. Briefly, 100 μL of whole mouse blood was mixed with 75 μL of FITC-conjugated anti-mouse CD4^+^ and PE-conjugated anti-mouse CD8^+^ monoclonal antibodies (BD Pharmingen, USA) and incubated in the dark at room temperature for 30 min. Afterwards, 2 mL of red blood cell lysis buffer (Roche, Germany) was added and incubated for 10 min. The reaction was terminated by adding 3 mL of PBS and the samples were centrifuged at 380 × *g* for 5 min. The cell pellet was washed twice with PBS, resuspended in 0.5 mL PBS and the stained blood cells were analyzed using BD FACSLyric flow cytometer and BD FACSuite software (BD Biosciences, USA).

### 2.9 Serum cytokine measurement

Peripheral blood samples were centrifuged at 2,000 rpm for 10 min at 4°C to obtain serum. Following the manufacturer’s instructions, 50 μL of mouse serum was analyzed using the Cytometric Bead Array (CBA) Mouse Inflammatory Kit (BD, Cat# 552364) to quantify cytokine levels, including IL-6, IL-12, IFN-*γ*, TNF-*α*, IL-10, and MCP-1. The samples were processed and analyzed using a BD FACSLyric flow cytometer with BD FACSuite software (BD Biosciences, USA).

### 2.10 Tissue processing and histological assessment

Spleen and liver samples were fixed in 10% neutral-buffered formalin for 48 h. The fixed tissues were then dehydrated using a series of alcohol and xylene treatments before being embedded in paraffin. The paraffin-embedded tissues were sectioned into 3-μm-thick slices using a rotary microtome and mounted onto microscopic slides. The sections were subsequently stained with hematoxylin and eosin (H&E) for histological analysis. For each sample, ten randomly selected areas were examined microscopically at ×100 magnification. Tables 1, 2 present the histological grading criteria for brucellosis in the liver and spleen of mice, respectively. The severity of microgranulomas, periportal inflammation, and necrosis was assessed using a 0–4 scale, as described in previous studies (21–23). The mean total score for each group was then compared.

**Table 1:** Histological grading criteria for brucellosis in the liver of mice.

Necrosis	Degree of Microgranuloma Formation	Degree of Periportal Inflammation	Severity Score
None	None	None	0
Mild focal necrosis	Minimal (1 / 100x field)	Minimal (<25% affected)	1
Mild to Moderate multifocal necrosis	Mild (2–3 / 100x field)	Mild (25–50% affected)	2
Moderate multifocal necrosis	Moderate (4–6 / 100x field)	Moderate (50–75% affected)	3
Severe multifocal to Coalescent necrosis	Marked (>7 / 100x field)	Marked (>75% affected)	4

**Table 2:** Histological grading criteria for brucellosis in the spleen of mice.

Degree of Microgranuloma Formation		Score
None		0
Minimal	(1 / 100x field)	1
Mild	(2–3 / 100x field)	2
Moderate	(4–6 / 100x field)	3
Marked	(>7 / 100x field)	4

### 2.11 Statistical analysis

All experiments were performed in triplicate and repeated in at least three independent experiments. The data were analyzed using Student’s *t*-test or one-way ANOVA in GraphPad Instat. Results were expressed as means ± standard deviation (SD). Differences with *p*-values < 0.05 *, *p* < 0.01 **, *p* < 0.001 *** were considered statistically significant.

## 3 Results

### 3.1 Effect of imipramine on cell viability and bacterial survivability

**Figure 1:**
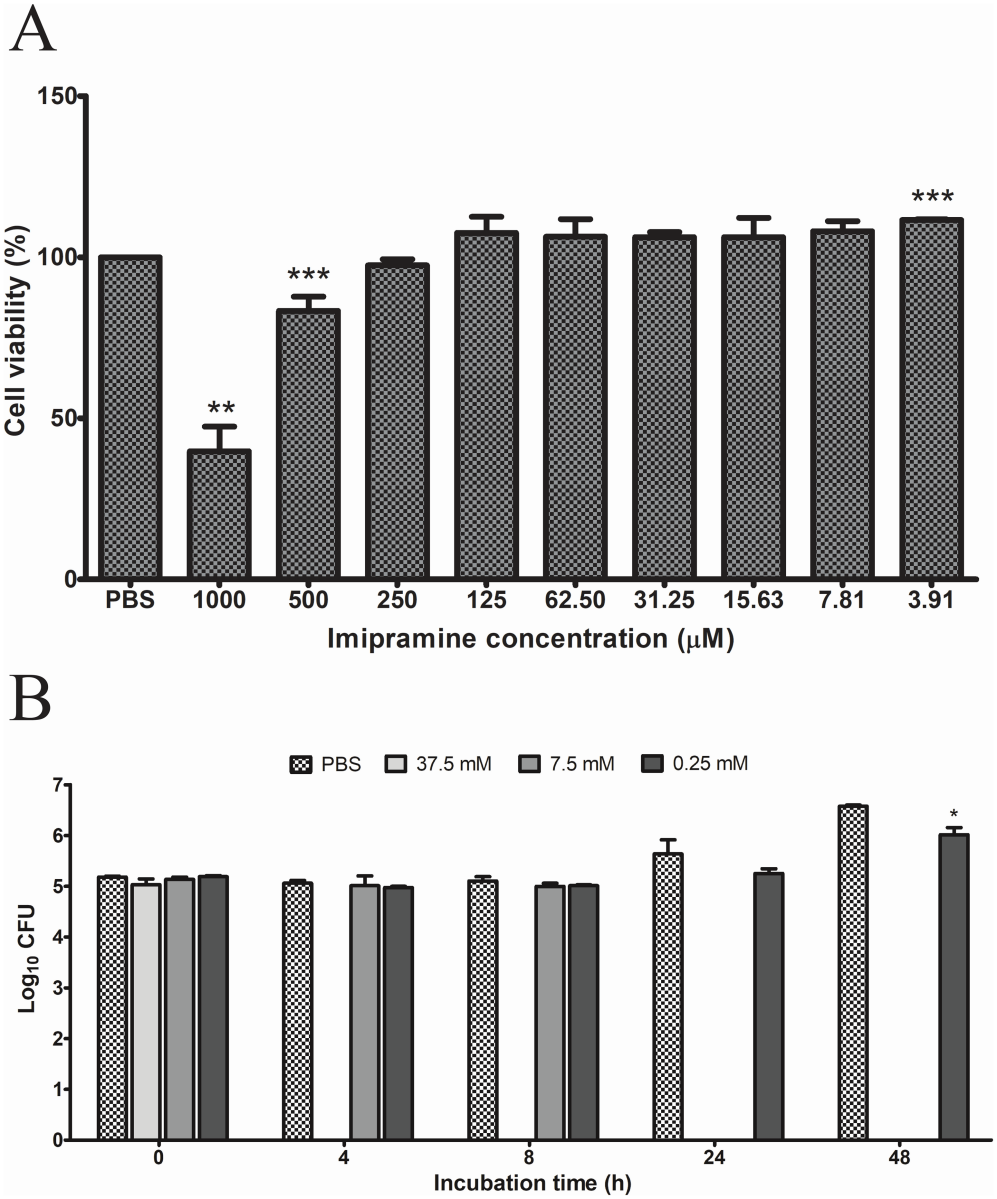
Effect of imipramine on RAW 264.7 cells and *B. abortus* growth. Cell viability was assessed using the MTT assay, identifying 250 μM as the highest non-cytotoxic concentration **(A)**. Imipramine exhibited a dose-dependent bactericidal effect against *B. abortus*, significantly reducing bacterial survival at 0.25 mM after 48 h **(B)**. Data are presented as mean ± SD of at least three replicates, with statistically significant differences from the control indicated by asterisks (*p < 0.05, **p < 0.01, ***p < 0.001).

RAW 264.7 cells incubated with different concentrations of ImiP exhibited reduced cell viability after 48 h of incubation with 1,000 μM and 500 μM of ImiP. In contrast, cell viability significantly increased at 3.91 μM compared to the control group. Treatment with 125 μM and 250 μM maintained cell viability at 107 and 97%, respectively, representing the highest non-cytotoxic concentrations. Therefore, 125 μM and 250 μM were selected for subsequent experiments (Figure 1A).

*B. abortus* were treated with 37.5 mM, 7.5 mM, and 0.25 mM ImiP to evaluate its bactericidal effect. Bacterial survival was assessed using triplicate wells for each concentration. As shown in Figure 1B, ImiP demonstrated a time- and dose-dependent effect against *B. abortus*. A marked reduction in CFU was observed in 37.5 mM group after 4 h and in the 7.5 mM group at 24 h post-treatment. The 0.25 mM group showed a significant CFU reduction at 48 h post-treatment compared to PBS, but to a lesser extent than the higher concentrations.

### 3.2 Effect of imipramine on internalization and intracellular growth of *Brucella abortus* in RAW 264.7 cells

**Figure 2:**
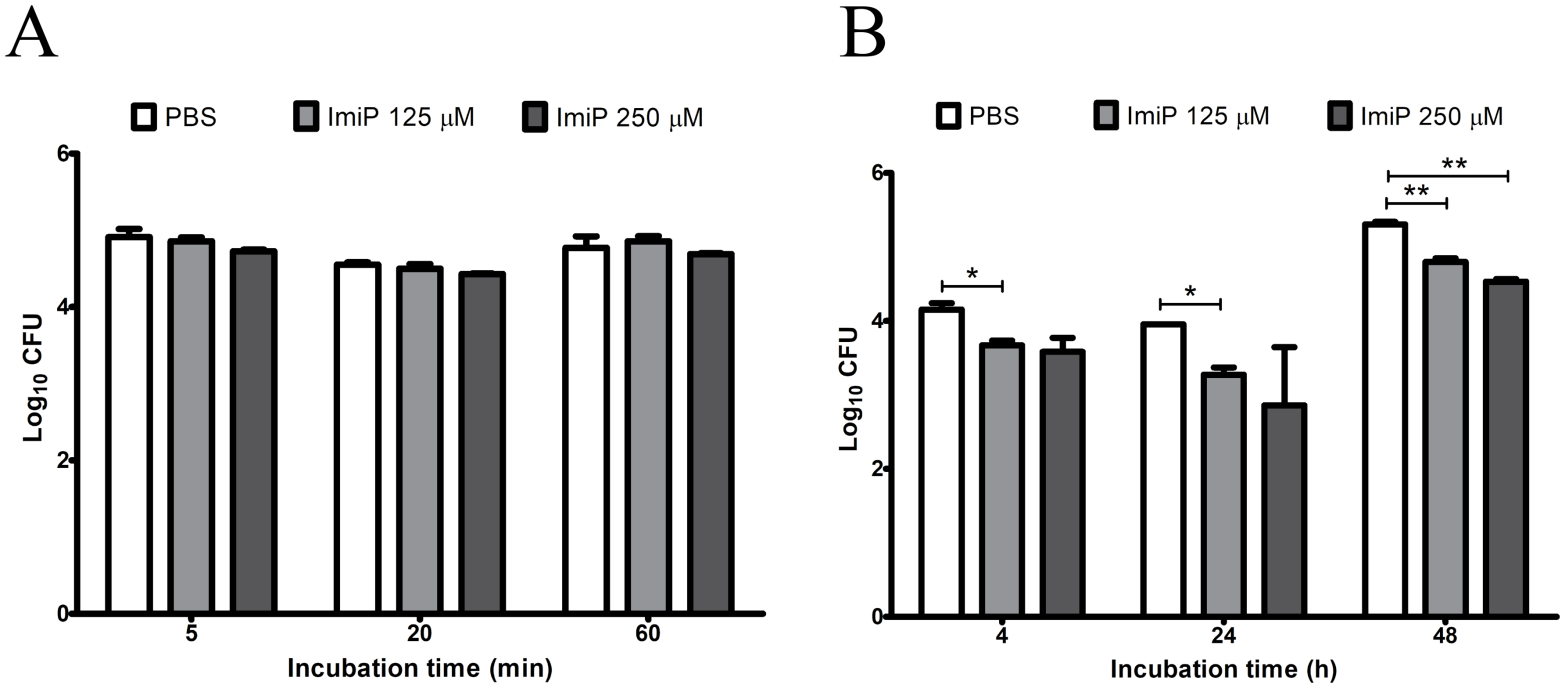
Effect of imipramine on *B. abortus* internalization and intracellular growth in murine macrophages. Bacterial internalization **(A)** and intracellular growth **(B)** were evaluated in infected macrophages treated with imipramine. Data represent the mean ± SD of triplicate samples from three independent experiments. Statistically significant differences from the control are indicated by asterisks (*p < 0.05, **p < 0.01).

Pre-treatment with 125 and 250 μM of ImiP did not affect the number of internalized bacteria in RAW 264.7 cells at 5, 20, and 60 min pi (Figure 2A). However, it significantly reduced the intracellular growth of *B. abortus* in cells treated with 125 μM ImiP at 4 (*p < 0.05), 24 (*p < 0.05) and 48 h (**p < 0.01) pi. Cells treated with 250 μM ImiP showed decreased intracellular bacterial growth at 48 h (**p < 0.01) pi (Figure 2B). Taken together, these findings suggest that ImiP treatment could interfere with the intracellular survival of *B. abortus* in macrophages.

### 3.3 Effect of imipramine on nitrite production in RAW 264.7 cells

**Figure 3:**
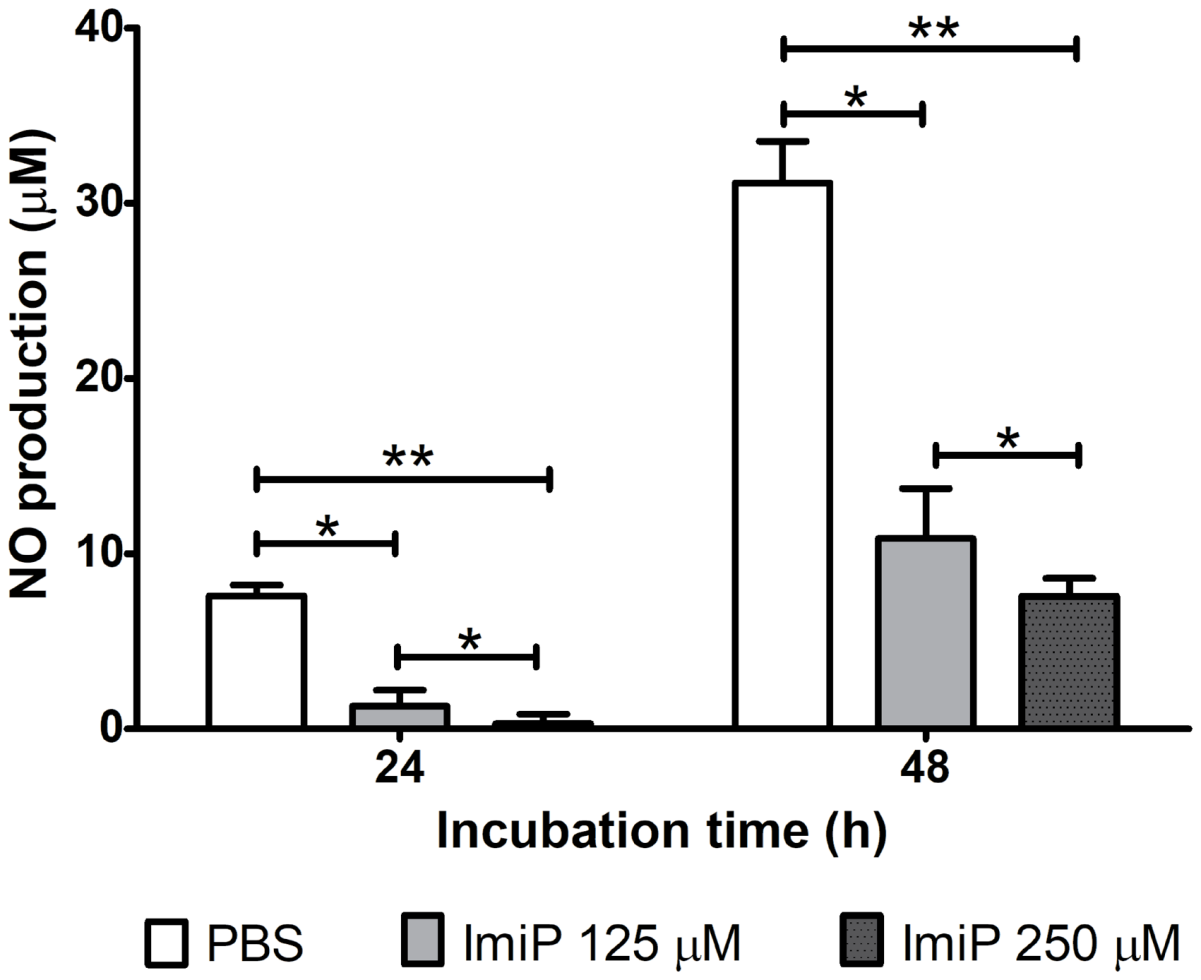
Imipramine reduces nitrite production in *B. abortus*-infected murine macrophages. Nitrite levels in RAW 264.7 cells decreased significantly after 24 and 48 h of incubation. Data are presented as mean ± SD of at least three replicates, with statistically significant differences from the control indicated by asterisks (*p < 0.05, **p < 0.01).

RAW 264.7 cells were infected and treated using the same method as the intracellular growth assay. After infection the cells were cultured in fresh medium with ImiP and gentamicin for 24, and 48 h. Nitric oxide (NO) plays a multifaceted role, particularly in host defense against pathogens, impacting both the progression and persistence of infections (24). ImiP-treated murine macrophages infected with *B. abortus* exhibited a significant, dose-dependent reduction in nitrite levels compared to the control at both 24 and 48 h pi (Figure 3).

### 3.4 Effect of imipramine on *Brucella abortus* infection in mice

**Figure 4:**
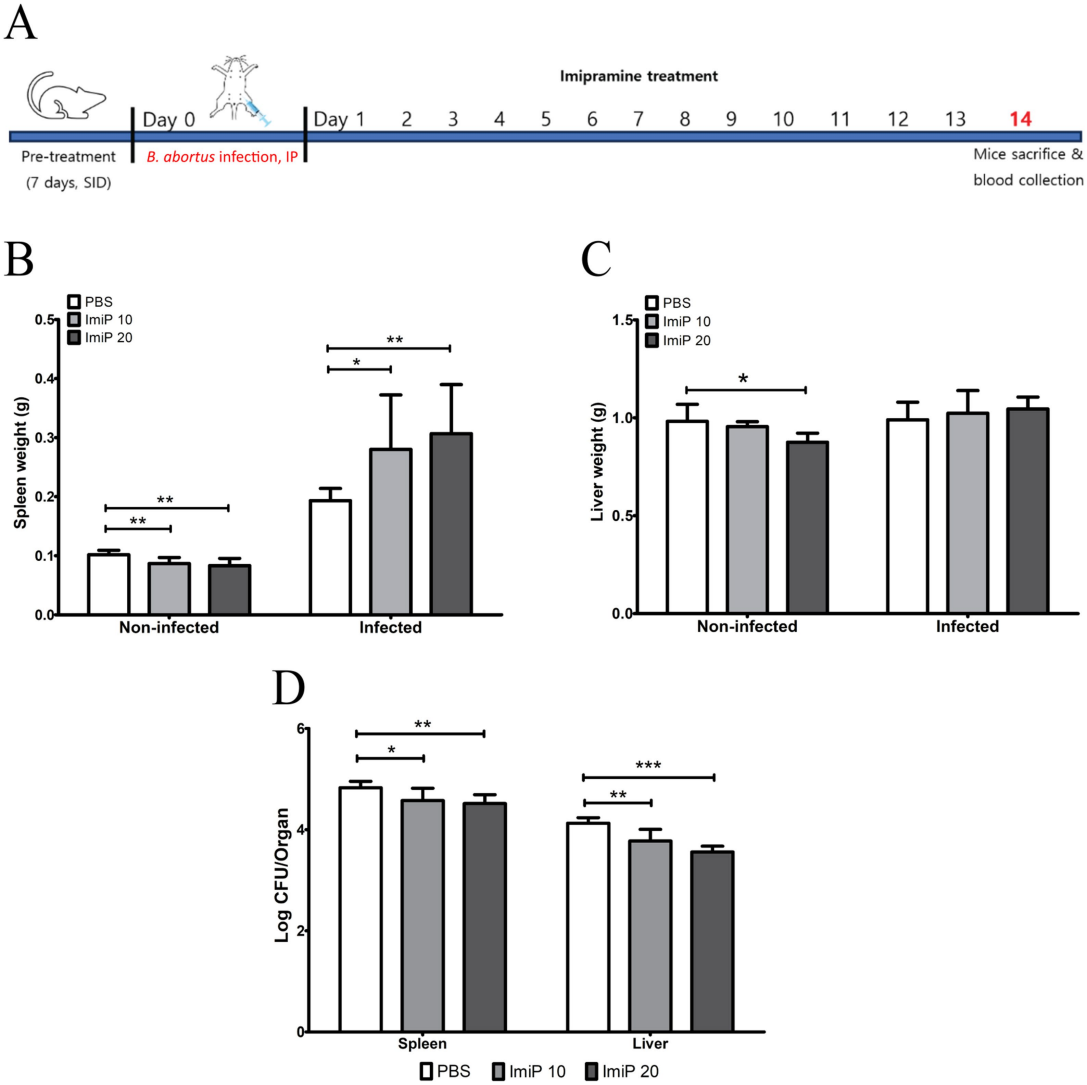
Protective effects of imipramine treatment against brucellosis. Following the experiment design **(A)**. BALB/c mice received oral imipramine (10 or 20 mg/kg/day) or PBS (control) for 1 week before *B. abortus* 544 infection, continuing treatment for 14 days. Spleen weight **(B)**, liver weight **(C)**, and bacterial load **(D)** in each organ were assessed 14 days post-infection. Data are presented as mean ± SD (n = 6 per group), with statistically significant differences from the control indicated by asterisks (* *p* < 0.05, ** *p* < 0.01, *** *p* < 0.001).

For the *in vivo* experiments, groups of six BALB/c mice were used (n = 6 per group). No clinical symptoms or mortality were observed throughout the treatment period. As shown in Figure 4A, BALB/c mice were orally treated with PBS (control), 10 mg/kg/day (low dose), or 20 mg/kg/day (high dose) of ImiP for 7 days, followed by intraperitoneal infection with *B. abortus*. The treatment was continued for 14 days then the mice were sacrificed to assess spleen and liver weights. In non-infected ImiP-treated mice, spleen weight significantly decreased in a dose-dependent manner compared to the control. However, infected mice treated with ImiP exhibited increased spleen weight at both doses (Figure 4B). A similar pattern was observed for liver weight, however only the non-infected mice treated with ImiP 20 showed a significant reduction (Figure 4C). Additionally, Imip treatment significantly reduced bacterial burden in the spleen, with log protection units of 0.25 for the low dose and 0.31 in the high dose group. Similarly, bacterial load in the liver decreased, with log protection units of 0.35 for the low dose and 0.57 in the high dose group (Figure 4D, Table 3).

**Table 3:** Protection against *B. abortus* in mice treated with ImiP.

Tissues	Treatment	Log_10_ CFU of bacteria (Mean ± SD)	Log protection	*p*-value^a^
Spleen	PBS	4.83 ± 0.13		
	ImiP 10 mg/kg	4.58 ± 0.24	0.25	*p* < 0.05
	ImiP 20 mg/kg	4.52 ± 0.17	0.31	*p* < 0.01
Liver	PBS	4.13 ± 0.11		
	ImiP 10 mg/kg	3.78 ± 0.23	0.35	*p* < 0.01
	ImiP 20 mg/kg	3.56 ± 0.11	0.57	*p* < 0.001

a Significant difference from PBS-treated mice were estimated by Student’s *t*-test.

### 3.5 Effect of imipramine on host immune response and cytokine production in RAW 264.7 cells

**Figure 5:**
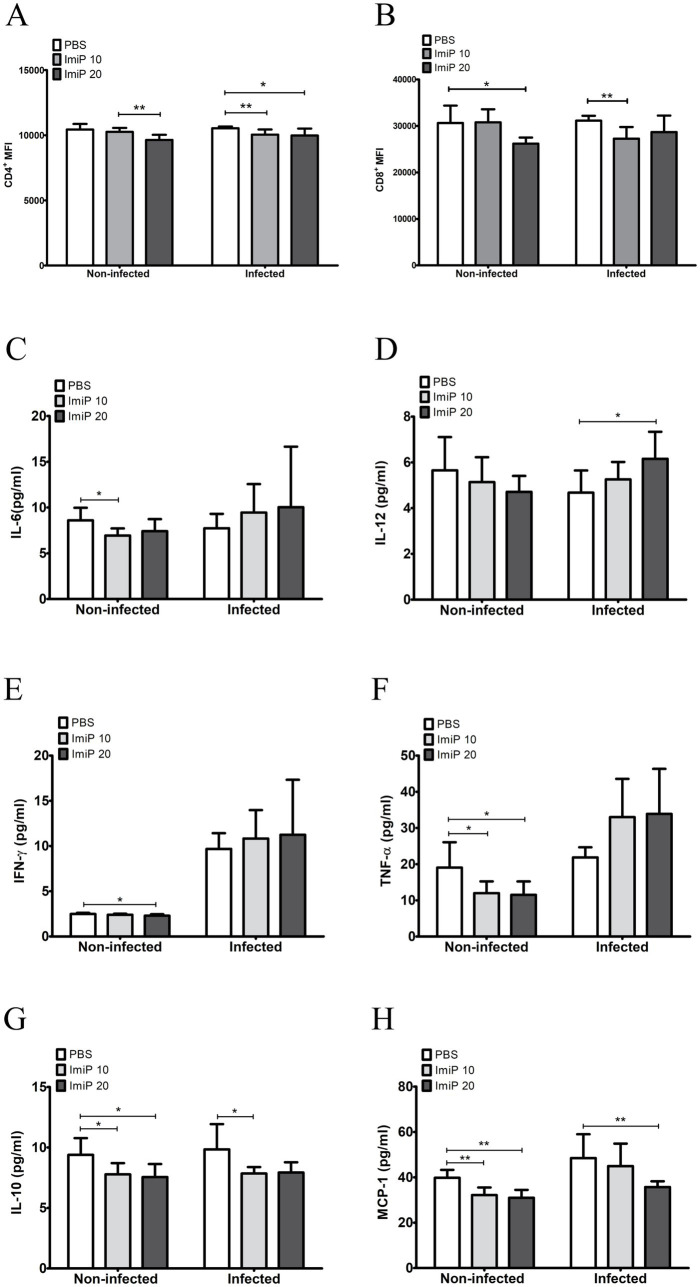
Imipramine modulates the immune response in *B. abortus*-infected mice. CD4^+^ **(A)** and CD8^+^ **(B)** mean fluorescence intensities, were measured using flow cytometry. Serum levels of IL-6 **(C)**, IL-12 **(D)**, IFN-γ **(E)**, TNF-*α* **(F)**, IL-10 **(G)**, and MCP-1 **(H)** were quantified using a CBA kit and flow cytometry. Data are presented as mean ± SD values of six mice per group, with statistically significant differences from the control indicated by asterisks (*p < 0.05, ** *p* < 0.01, *** *p* < 0.001).

To evaluate the effect of ImiP on the host immune response, CD4^+^ and CD8^+^ cell populations were analyzed, along with cytokine measurement using a CBA kit. At 14 days pi, CD4^+^ levels decreased in both non-infected and infected groups. In the non-infected group, no significant difference was observed between the treated and control groups. However, a significant reduction was noted in the group treated with the higher dose of ImiP compared to the low dose group. In the infected group, CD4^+^ levels significantly decreased at both treatment doses compared to the control. Similarly, CD8^+^ levels also declined, with a significant reduction in the non-infected mice treated with 250 μM ImiP and in infected mice treated with 125 μM ImiP (Figures 5A,B).

The CBA kit quantified the level of six cytokines which were IL-6 (Figure 5C), IL-12 (Figure 5D), IFN-*γ* (Figure 5E), TNF-*α* (Figure 5F) while IL-10 (Figure 5G) and MCP-1 (Figure 5H). The cytokine analysis of peripheral blood revealed that infected mice treated with ImiP significantly increased levels of IL-12 while IL-10 and MCP-1 levels decreased. These findings suggest that ImiP possesses immunomodulatory properties that may enhance host defenses.

### 3.6 Impact of imipramine on *Brucella*-induced hepatic lesions

**Figure 6:**
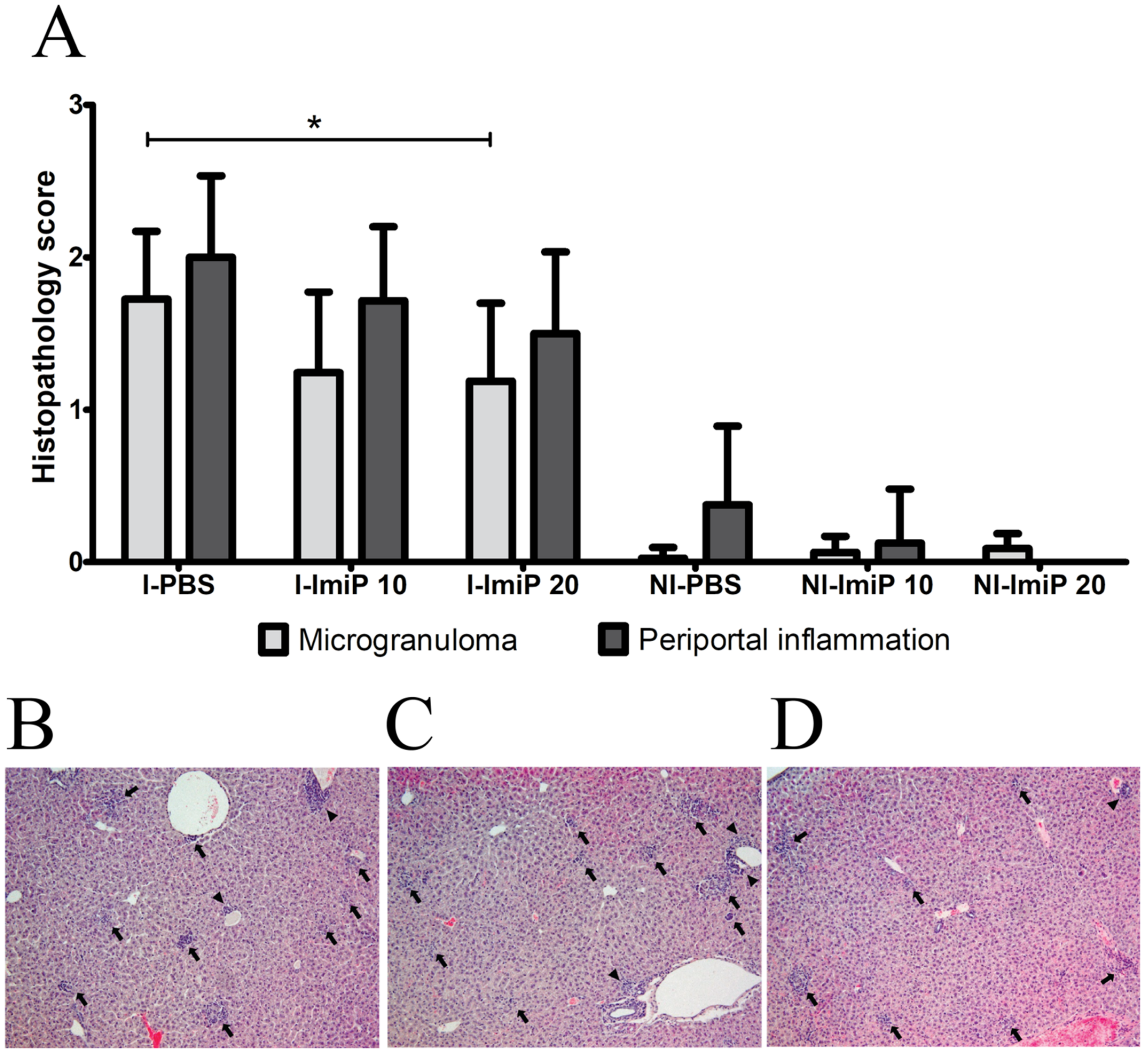
Histopathological evaluation of the liver in *B. abortus*-infected mice. The presence of microgranulomas and periportal inflammation was evaluated in infected (I) and non-infected (NI) groups treated with imipramine or control treatments **(A)**. Microscopic examination at ×100 magnification revealed periportal inflammation (arrowheads) and microgranulomas (arrows) in infected mice treated with PBS **(B)**, imipramine 10 mg/kg **(C)**, and 20 mg/kg **(D)**. Data are presented as the mean ± SD of six mice per group, with statistically significant differences from the control indicated by asterisks (*p < 0.05).

Microscopic examination of stained liver and spleen sections revealed no significant lesions in the spleen. In the liver, the non-infected group treated with ImiP exhibited minimal microgranuloma formation and periportal inflammation. Conversely, these lesions were reduced in *Brucella*-infected groups receiving ImiP treatment, with microgranuloma formation lower in the high dose group compared to low dose and control group (Figure 6A). Notably, necrosis was absent in all treated liver samples. Representative histological liver samples from infected groups treated with PBS (Figure 6B), 10 mg/kg ImiP (Figure 6C), and 20 mg/kg ImiP (Figure 6D) further confirmed the reduction in microgranuloma formation and periportal inflammation, supporting the histological assessment.

## 4 Discussion

The ability of *Brucella* spp. to survive within host cells and evade immune responses makes these bacteria difficult to eradicate. Therefore, exploring treatments that target their survival mechanisms is crucial, especially given the limited treatment options currently available (5). Several studies have highlighted the therapeutic benefits of imipramine (ImiP) beyond its primary role in the nervous system. Biswas et al. (25) reported its anti-inflammatory properties, demonstrating that ImiP significantly reduced silicosis in a mouse model. Additionally, ImiP has shown effectiveness in treating infectious diseases, such as *Leishmania* infections (13). As well as, potential anti-cancer properties including the inhibition of metastasis and modulation of cell death pathways (26, 27). Nevertheless, the use of ImiP in treating infectious diseases presents certain challenges. In humans, ImiP is associated with various adverse effects such as dry mouth, constipation, blurred vision, tachycardia and seizures. These side effects may pose significant risks, particularly for immunocompromised patients. Moreover, overdose with TCAs can be life-threatening, necessitating careful monitoring to prevent toxicity (28). Even though, it is a relatively low-cost generic drug, the need for medical supervision and the risk of drug interactions may increase the overall burden of treatment (29). These factors must be considered when evaluating the suitability of ImiP as a therapeutic option for infectious diseases.

Although previous research indicated that ImiP has no *in vitro* efficacy against *Brucella* spp. (30), this study demonstrates its potential therapeutic role against brucellosis. Murine macrophages (RAW 264.7 cells) were used due to their ability to carry out both pinocytosis and phagocytosis. (31). ImiP exhibited a direct bactericidal effect on *B. abortus* 544 (Figure 1B) and inhibited its intracellular growth (Figure 2B). ImiP belongs to the group of cationic amphiphilic drugs (CADs). These drugs can freely diffuse across cell membranes and accumulate in macrophage lysosomes. This accumulation may alter cholesterol and lipid metabolism, key factors in regulating membrane integrity (32). ImiP is also an acid sphingomyelinase (ASMase) inhibitor, leading to decreased ceramide production and disrupted lipid raft formation (33). Since *Brucella* utilizes lipid rafts for macrophage entry, this could impair bacterial uptake. Although ImiP did not decrease bacterial internalization in this study, it successfully inhibited the intracellular growth of *Brucella*. A possible mechanism is that ASMase inhibition alters lysosomal lipid composition. Leading to impaired fusion of *Brucella*-containing vacuole (BCV) with the endoplasmic reticulum (ER) compartments, which are essential for bacterial replication. Additionally, ImiP may enhance lysosomal fusion with BCVs leading to bacterial degradation (34–36). Furthermore, ImiP treatment reduced NO production in infected macrophages (Figure 2C). NO is a key mediator of inflammation and intracellular pathogen control (37). The significant reduction of NO aligns with the reported immunomodulatory properties of ImiP. By modulating the production of NO, ImiP may help balance bacterial replication control while minimizing inflammation (38, 39).

To evaluate its safety and efficacy *in vivo*, mice were orally treated with two doses of ImiP (10 mg/kg/day and 20 mg/kg/day). Both doses significantly reduced bacterial load in the liver and spleen. Histological analysis showed minimal microgranuloma formation and periportal inflammation in non-infected mice. In contrast, infected mice treated with ImiP exhibited reduced lesion in a dose-dependent manner. These results are consistent with the research conducted by Ayad et al. (17), who reported that the hepatotoxic effect of ImiP is dose-dependent, resulting in swelling of centrilobular hepatocytes and vascular congestion. Similarly, microgranuloma formation in the liver is a common lesion observed in brucellosis as part of the tissue response to infection.

ImiP treatment significantly affect cytokine levels and CD4^+^ and CD8^+^ T cell populations. In *Brucella*-infected mice treated with ImiP, IL-12 levels increased. IL-12 is crucial for controlling intracellular infections. Other pro-inflammatory cytokines essential in controlling brucellosis include IL-6, IFN-*γ*, and TNF-*α* (40, 41). IL-12 also bridges the innate and adaptive immunity by stimulating T cells and natural killer (NK) cells to produce IFN-γ, which promotes the differentiation of naïve T cells into Th1 cells. This Th1 response is crucial for effective bacterial clearance, as it enhances macrophage activation and promotes the destruction of intracellular pathogens (42).

In contrast, IL-10 and MCP-1 levels decreased following ImiP treatment. IL-10 is an anti-inflammatory cytokine that suppresses Th1 activity and limits pro-inflammatory cytokine production. MCP-1 facilitates the recruitment and movement of monocytes and macrophage. In *Brucella* infection, high IL-10 levels can contribute to pathogen persistence by dampening the immune response and inhibiting macrophage bactericidal activity (22, 43, 44). The reduction in IL-10 suggests that ImiP treatment shifts the immune response toward a more pro-inflammatory state, enhancing the host’s ability to clear *Brucella* infection.

The inverse relationship between increased IL-12 and decreased IL-10 suggests that ImiP enhances host defense mechanisms. It promotes a Th1-driven immune response while suppressing regulatory pathways that could favor bacterial survival. Additionally, although CD4^+^ and CD8^+^ T cell levels are linked to protection against *B. abortus* strain 19 in BALB/c mice (45). However, in this study, the MFI of these markers decreased, suggesting that ImiP treatment may suppress T-cell activation. This aligns with a previous study reporting that TCAs, including imipramine, induce apoptosis in CD4^+^ and CD8^+^ human T lymphocytes (46). These findings highlight the complex regulatory interactions between innate and adaptive immunity influenced by ImiP (47).

In summary, this study demonstrates the immunomodulatory potential of ImiP in strengthening host defenses against *B. abortus* 544 infections. ImiP exhibits bactericidal effects, inhibits intracellular bacterial growth, and reduces NO levels, suggesting its role in controlling *Brucella* infection. It also promotes a Th1 shift (increased IL-12 and decreased IL-10), which correlates with reduced bacterial burden in infected mice. Furthermore, ImiP treatment decreased CD4^+^ and CD8^+^ MFI, indicating a regulation of T cell activation. As ImiP demonstrated direct inhibitory effects on *B. abortus in vitro*, the immune effects observed *in vivo* may partly result from reduced bacterial load. These findings indicate that ImiP maintains an effective yet controlled immune response, enhancing bacterial clearance while preventing excessive immune activation and inflammation-associated tissue damage. However, further research is needed to understand how ImiP influences intracellular bacterial clearance, T-cell regulation, and its long-term safety and efficacy. Such studies will be essential for optimizing brucellosis treatment strategies and mitigating the global burden of zoonotic diseases.

## Data Availability

The original contributions presented in the study are included in the article/supplementary material, further inquiries can be directed to the corresponding author.
